# Notes

**Published:** 1901-09

**Authors:** 


### NOTES.

Dr. J. O. Roberts, of Greenville, Miss., reports a most virulent outbreak of anthrax in some parts of the Mississippi Valley. Mississippi, Louisiana, and Arkansas have all lost to a greater or less extent. One county alone in Mississippi has already lost more than $25,000 worth of live-stock. Dr. Roberts has found many germs in the stomach of the flies, which are mainly responsible for the rapid spread of the disease.

From the beginning of the Boer war to May 31 last the British Government landed in Africa 172,985 horses and 80,723 mules, or a total of 253,708. A count on May 11th last showed a total in active service with the army of 185,000—a loss of 68,708 during the war.

Nashville, Tenn., falls in line with the large cities of the East and North, and offers the horse-admiring public an "up-to-date" horseshow. The management has promised a complete show in every particular. Dr. William C. Rayen has been appointed to officiate as veterinarian.

